# Examining neuroanatomical correlates of win-stay, lose-shift behaviour

**DOI:** 10.1007/s00429-025-02901-z

**Published:** 2025-02-27

**Authors:** Matt Westerman, Glyn Hallam, Alex Kafkas, Holly D. H. Brown, Chris Retzler

**Affiliations:** 1 School of Health Sciences, Division of Psychology, Communication & Human Neuroscience, University of Manchester, G.010 Dover Street Building, Manchester, M13 9PL UK; 2 Department of Psychology, The University of Huddersfield, Huddersfield, UK; 3 School of Education, Language and Psychology, York St John University, York, UK; 4 School of Psychology, University of Leeds, Leeds, UK

**Keywords:** Decision-making, Win-stay, Lose-shift, Grey matter volume, White matter volume, Occipital cortex

## Abstract

This study aimed to better understand the neuroanatomical correlates of decision-making strategies, particularly focusing on win-stay and lose-shift behaviours, using voxel-based morphometry (VBM) in a large cohort of healthy adults. Participants completed a forced-choice card-guessing task designed to elicit behavioural responses to rewards and losses. Using this task, we investigated the relationship between win-stay and lose-shift behaviour and both grey matter volume (GMV) and white matter volume (WMV). The frequency of win-stay and lose-shift behaviours was calculated for each participant and entered into VBM analyses alongside GMV and WMV measures. Our results revealed that increased lose-shift behaviour was associated with reduced GMV in key brain regions, comprising of the left superior temporal gyrus, right middle temporal gyrus, and the bilateral superior lateral occipital cortices. Interestingly, no significant associations were found between GMV or WMV, and win-stay behaviour. These results suggest that specific regions within the temporal and occipital lobes may be involved in modulating decision-making strategies following negative outcomes. Further analyses revealed that increased lose-shift behaviour was also associated with increased WMV in the left superior temporal gyrus. The absence of significant findings in relation to win-stay behaviour and the differential involvement of brain structures in lose-shift responses indicate that decision-making in the face of losses may involve distinct neuroanatomical mechanisms compared to decision-making following wins. This study advances our understanding of the structural brain correlates linked to decision-making strategies and highlights the complexity of brain-behaviour relationships in choice behaviour.

**Supplementary Information:**

The online version contains supplementary material available at 10.1007/s00429-025-02901-z.

## Introduction

Human decision-making is often guided by the outcomes of previous decisions (Rushworth et al. 2011). To make appropriate and subjectively advantageous decisions, we adapt our decision-making strategies based on whether a similar prior decision elicited a positive or negative outcome (Donahue et al. 2013; Neville et al. 2021). Generally, if a decision results in a positive outcome (i.e. it is rewarding), the individual is more likely to repeat the decision (i.e., win-stay behaviour; Nowak and Sigmund 1993; Forder and Dyson 2016; Zhang et al. 2023). However, if a decision results in a negative outcome (i.e., a loss that is subjectively aversive or unpleasant), the individual is more likely to avoid using a similar decision strategy in the future. (i.e., lose-shift behaviour; Nowak and Sigmund 1993; Donahue et al. 2013; Wang et al. 2014; Deng et al. 2016; Forder and Dyson 2016; Gutiérrez-Roig et al. 2016; Zhang et al. 2021; Chu et al. 2022). This process of updating decision strategies is an example of adaptive behavioural learning, where individuals will adjust their actions based on prior experiences to optimise outcomes. Adaptive behavioural learning has been identified as a core mechanism underlying behavioural flexibility and neural processes that facilitate decision-making in dynamic environments (O’Reilly 2013; Schulz et al. 2019). Variations in how individuals respond to losses may reflect differences in adaptive capacity and could be a precursor to the pervasive use of aberrant decision-making strategies, such as those associated with gambling disorder (Bechara 2005; Brevers et al. 2013; Diekhof et al. 2008). While adaptive decision-making is fundamental to behavioural flexibility, less is known about how choice behaviour operates in tasks that lack a clearly defined optimal decision strategy, such as the one employed in this study.

When the outcomes of our decisions are uncertain, we can employ decision-making strategies such as win-stay and lose-shift (WSLS). This strategy can be applied to games like ‘rock, paper, scissors’, a zero-sum game where there is no clear optimal decision strategy (Paulus et al. 2005; Forder & Dyson 2016). It is thought that playing randomly is optimal under these circumstances as it diminishes the opponent’s ability to predict a sequence in a series of choices (i.e., consistently choosing “rock” after a win) but evidence suggests that humans struggle to play completely randomly (Forder and Dyson 2016) and instead rely on strategies such as WSLS (Lin et al. 2007; Bonawitz et al. 2014; Forder and Dyson 2016; Gutiérrez-Roig et al. 2016). Forder and Dyson (2016) showed that increasing win and loss amounts on a rock, paper, scissors task can increase win-stay and reduce lose-shift behaviour, respectively. In addition, they showed that decision-making following a win was characterised as slow and flexible, with a behavioural increase in the use of the win-stay strategy and neural modulation of feedback-related negativity and stimulus-preceding negativity components. In contrast, decision-making following a loss was described as relatively fast and inflexible, with a failure to utilise the lose-shift strategy and a lack of significant neural modulation (Forder and Dyson 2016). These findings suggest that the WSLS heuristic may not be a singular decision strategy but rather a dissociation between the underlying cognitive and neural processes (Forder and Dyson 2016).

WSLS behaviours are not limited to paradigms like “rock, paper, scissors” (e.g., Foder and Dyson, 2016) but are evident in real-world scenarios. For instance, a business might continue investing in a profitable venture (win-stay) while discontinuing a failing strategy to minimise losses (lose-shift). Similarly, in everyday life, a driver may stick to a frequently used route that avoids traffic (win-stay) or take an alternative path after encountering congestion (lose-shift). These behaviours highlight how WSLS strategies enable individuals to optimise outcomes in dynamic and uncertain environments. By investigating the neuroanatomical correlates of these behaviours, the present study provides insights into the structural brain mechanisms that support adaptive decision-making in response to real-world challenges.

WSLS behaviours have traditionally been linked to reward systems involving the striatum, anterior cingulate cortices and orbitofrontal cortices, which play critical roles in reward evaluation, reinforcement learning and decision-making (Haber and Knutson 2010; Peters and Büchel 2010). Such regions are involved in approach-avoidance behaviour, where approach behaviour is driven by positive outcomes (e.g., win-stay) and avoidance behaviour is driven by negative outcomes (e.g., lose-shift) (Amemori et al. 2015; Forder and Dyson 2016; LeDoux and Daw 2018). A well-established functional neuroimaging literature has demonstrated that receiving a reward is associated with increased activation within the mesolimbic dopaminergic system, particularly the ventral striatum (Balleine et al. 2007; Delgado et al. 2000; Haber and Knutson 2010; O'Doherty et al. 2017; Pessiglione and Delgado 2015). Importantly, activation within the ventral striatum displays a differential response to reward and loss outcomes, with anterior regions activated by rewards and posterior regions by losses (Delgado et al. 2000; Seymour et al. 2007; Soares-Cunha et al. 2016). However, emerging evidence suggests that the neural basis of WSLS behaviour may extend beyond traditional reward systems. For instance, decision-making following a loss has been associated with increased activation in the precuneus, superior temporal gyrus (Dong et al. 2015), insula (Dong et al. 2014; Xue et al. 2010), frontoparietal network (Xue et al. 2010), and regions such as the cingulate cortex and inferior frontal gyri (Dong et al. 2014; Xue et al. 2010).

While there is good evidence for the use of WSLS strategies in human and animal behaviour (Nowak and Sigmund 1993; Donahue et al. 2013; Wang et al. 2014; Deng et al. 2016; Forder and Dyson 2016; Gutiérrez-Roig et al. 2016; Zhang et al. 2021; Chu et al. 2022), the relationship between such strategies and brain structure is not fully understood. In humans, reduced grey matter volume (GMV) in the insula and prefrontal cortex have been associated with loss aversion (Markett et al. 2016) and heightened reward sensitivity, respectively (Adrián-Ventura et al. 2019), whilst increased GMV of the amygdala has been connected to greater loss sensitivity (Adrián-Ventura et al. 2019). In addition, research has highlighted the importance of considering the value of wins and losses, as this can influence the use of WSLS strategies (Sacré et al., 2017; Srihaput et al. 2020). Taken together, this research provides initial evidence that brain structure may be associated with internal representations of reward or loss outcomes.

The neural correlates of WSLS behaviour have been studied in rodents with some studies showing that lesions within the rodent striatum (dorsal medial and dorsal lateral) reduce lose-shift behaviour (Skelin et al. 2014; Thapa and Gruber 2018) but not win-stay behaviour, suggesting that specific structural abnormalities may impact lose-shift behaviour. In humans, reduced GMV within the thalamus and associated nuclei have been associated with increased use of WSLS behaviour in methamphetamine dependence (Harlé et al. 2015). Within WSLS paradigms, responses to rewards and losses in healthy populations have been associated with increased neural activity compared to baseline (Forder and Dyson 2016; Xue et al. 2012). Additionally, greater activation in the frontal pole and posterior cingulate cortex has been associated with the increased use of WSLS behaviour (Xue et al. 2012). WSLS behaviour may be a default strategy due to its associations with reinforcement learning (Barraclough et al. 2004; Huang et al. 2019), which is driven by the impact of rewards and losses (Forder and Dyson 2016). Therefore, WSLS behaviour may be related to a sensitivity to rewards and losses, which has been previously associated with GMV (Adrián-Ventura et al. 2019). In turn, GMV has previously been associated with the gambler’s fallacy (Huang et al. 2019), which is the mistaken belief that past independent events affect future outcomes. In healthy individuals, the gambler’s fallacy has been linked with increased GMV within the bilateral ventral striatum and orbitofrontal cortex and reduced GMV in the frontal pole, anterior cingulate and left medial temporal lobe (MTL) (Huang et al. 2019). WSLS strategy and the gambler’s fallacy are underpinned by different cognitive processes. The WSLS involves recognising and adjusting to actual outcome patterns, while the gamblers’ fallacy involves erroneous beliefs about the connection of independent events. However, both strategies are reflective of cognitive processes that monitor outcomes. As demonstrated by a recent investigation (Michel et al., 2024), it is important to consider both GMV and white matter volume (WMV) when measuring aspects of cognition, as such metrics provide distinct yet complementary evidence related to cognitive performance; specifically processing power (grey matter) and efficient communication (white matter). Given that the gambler’s fallacy has been associated with GMV in healthy individuals (Huang et al. 2019), we expect WSLS behaviour to also be associated with both GMV and WMV in healthy individuals.

White matter is essential for neural connectivity, facilitating efficient communication between brain regions and enabling coordinated cognitive and behavioural regulation (Fields 2008; Filley 2010). Recent studies have highlighted the significance of WMV in predicting cognitive performance and decision-making outcomes. For example, Michel et al. (2024) found that WMV was the strongest predictor of cognitive performance, even when compared to more microstructural measures such as diffusion tensor imaging (DTI), emphasising its role as a robust macrostructural marker of brain function. Other studies have reported similar findings, linking WMV to temperament and character in young females (Van Schuerbeek et al. 2011), cognitive performance in older adults (Fletcher et al. 2013; Feng et al. 2013), and both processing speed (Magistro et al. 2015) and deception (Yang et al. 2005). Together, these findings underscore the importance of examining both GMV and WMV in the context of Voxel-based Morphometry (VBM) based protocol/analysis to provide a comprehensive understanding of brain-behaviour relationships.

WSLS behaviours reflect decision-making strategies that rely on immediate feedback to guide future choices, distinguishing them from the more complex risk-taking behaviours observed in gambling contexts (Worthy et al. 2013). While WSLS involves a relatively straightforward evaluation of outcomes—repeating decisions after wins and altering strategies following losses—gambling behaviours often incorporate subjective risk preferences, probabilistic reasoning, and emotional biases, such as overconfidence or loss aversion (Kahneman & Tversky 1979; Tom et al. 2007). For instance, a gambler may persist in betting despite repeated losses due to cognitive biases such as the “gambler's fallacy,” which assumes that a win is due after a series of losses (Croson and Sundali 2005). This contrasts with the lose-shift behaviour, which encourages individuals to change their approach to minimise losses. Additionally, while WSLS focuses on discrete, immediate decisions, gambling often involves long-term strategies and anticipation of uncertain outcomes, further complicating the neural and behavioural mechanisms involved (Clark et al. 2009). Understanding these distinctions helps position WSLS within a broader framework of decision-making strategies and highlights its role in adaptive behaviours that are less influenced by risk tolerance or irrational beliefs.

The current study investigated the neuroanatomical correlates of choice behaviour, specifically WSLS behaviour, in contexts where optimal decision-making processes are absent. A prior investigation found that whole brain GMV was associated with “switching” behaviour (changing response selection) regardless of the outcome of a previous trial (Sun et al. 2018), with a positive relationship between the frequency of response-switching and GMV of the posterior cingulate gyrus, left insula and frontal pole, and a negative correlation with GMV of the MTL and right insula. However, Sun et al. (2018) did not investigate the effect of the valence of a previous outcome (reward or loss) on this relationship. We, therefore, aimed to build upon the findings of Sun et al. (2018) to determine whether whole-brain GMV is associated with general switching behaviour and to identify associations between GMV and WSLS behaviour.

On the basis of the literature reviewed here, three hypotheses were formulated which were pre-registered on the Open Science Framework (https://osf.io/e563n) but have since been refined to improve specificity. We predicted that: 1. There would be a significant positive correlation between GMV in the frontal pole, posterior cingulate and left insula, and the overall tendency to switch response option, 2. There would be a significant negative correlation between GMV in the MTL and right insula, and the overall tendency to switch response options, and 3. There would be a significant correlation between GMV of the right insula and MTL, and frequency of switching after loss trials.

## Method

### Participants

We obtained structural MRI scans that were collected by the Human Connectome Project (HCP; Van Essen et al. 2013) from 1113 participants originally released within the 1200 subjects release dataset on March 1st, 2017. Informed consent had previously been obtained for all participants (consent procedure outlined in Van Essen et al. 2013). 889 participants had both T1-weighted MRI scans and behavioural data from the card value task. A further 16 participants were excluded due to incomplete behavioural data, three were missing a full T1 structural scan, one participant was discounted for having all response times faster than 200ms, and a further 19 were excluded as they did not engage with the task (pressed only one response throughout). The final sample size was, therefore, 850 participants. Participants were aged between 21 and 36 years of age (M = 26.92, SD = 3.49) of which 390 were male (Median age for all participants = 27.5). Handedness was also assessed as part of the restricted HCP dataset using the Edinburgh Handedness Inventory (Oldfield 1971). (EHI). Based on their EHI scores, participants were classified into five handedness groups—strong right-handed (+ 100 to + 80; n = 478), moderate right-handed (+ 79 to + 40; n = 262), ambidextrous (+ 39 to − 39; n = 56), moderate left-handed (− 40 to − 79; n = 35), and strong left-handed (− 80 to − 100; n = 19). Ethical approval for this study was granted by the School of Human and Health Sciences – School Research Ethics and Integrity Committee (SREIC) at the University of Huddersfield. All participants included in the HCP Healthy Young Adult 1200 dataset had corrected to normal vision and had no significant history of psychiatric disorder, substance abuse, neurological or cardiovascular disease, head injuries or genetic disorders. For a full list of exclusion criteria, please see supplementary data 01 in Van Essen et al., (2013). All data utilised in the current study were reviewed by a radiologist at the HCP (see Marcus et al. 2013). In addition, each scan was checked for artefacts by an experienced member of the research team. The current study was preregistered on the Open Science Framework (https://osf.io/e563n/).

### Card guessing paradigm

**Fig. 1:**
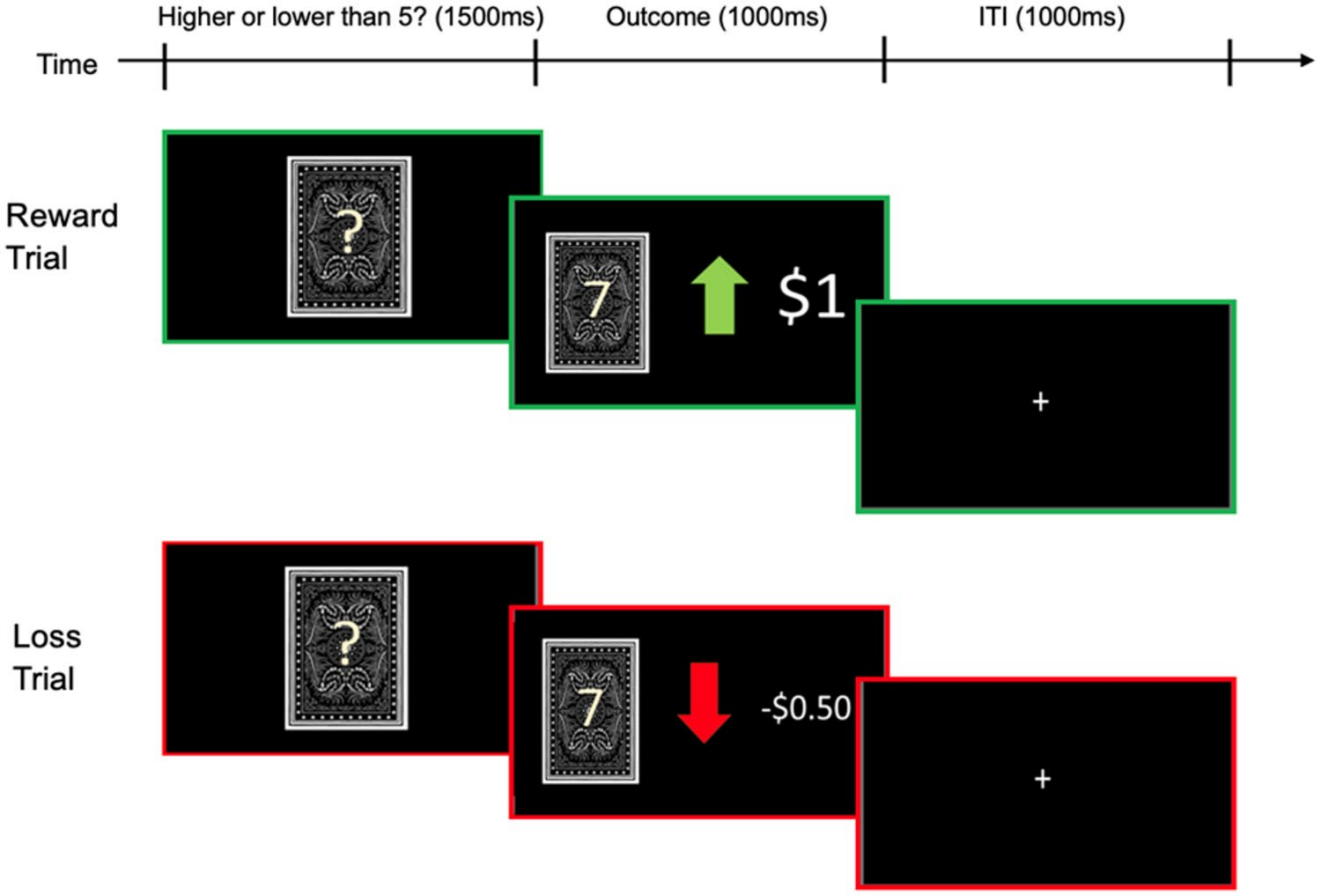
Card guessing task collected as part of the HCP data set. The top panel depicts a ‘reward’ trial. The lower panel depicts a ‘loss’ trial. Participants were required to guess whether the value of a card was higher or lower than 5. If correct, participants received a reward of $1. If incorrect, they incurred a loss of $0.50. *Note:* This figure has been created for illustrative purposes and is not a direct visual example of the task

The current study utilised data collected from an adapted version of the Delgado et al. (2000) reward paradigm in which participants guessed whether the hidden number of a playing card (i.e., 1–9) was greater or less than the value of 5. Each trial began with a question mark (1500ms), which cued participants to make a guess as to the value of a single card using one of two buttons, one on the right indicating “higher” and one on the left indicating “lower”. The outcome of each trial was predetermined, categorising trials into either a ‘reward’ trial (correct guess; + $1), a ‘loss’ trial (incorrect guess; −$0.50) or a neutral event (i.e., the card was 5; no gain or loss). Feedback on whether the response was correct or incorrect was then presented for 1000 ms, followed by an intertrial interval in which a fixation (“ + ”) was presented for 1000 ms. The task was presented in blocks of eight trials that were either mostly reward or mostly loss but still contained a mix of trial types. In each of the two runs, there were two mostly reward and two mostly loss blocks interleaved with four fixation blocks (duration 15 s). Each participant completed a total of 64 trials. Trials on which participants changed their response (i.e., higher to lower or lower to higher) were identified as ‘switch’ trials and were further split into switches following reward and switches following loss. Neutral trials were infrequent in occurrence and were excluded from analyses (6 trials in total). Any trial with a response quicker than 200ms was also excluded from the analyses (Fig. 1).

### Structural MRI data collection

Structural MRI scans were acquired at Washington University in St Louis between August 2012 and October 2015 on a Siemens 3.0 T “Connectome Skyra” (Siemens AG, Erlanger, Germany), using a 32-channel head coil. T1-weighted images were acquired using 3D, gradient echo pulse sequence (MPRAGE) with a resolution of 0.7 × 0.7x 0.7 mm^3^ isotropic (FOV = 224 × 224, matrix = 320 × 320, 256 sagittal slices; TR = 2400 ms and TE = 2.14 ms). For further information on quality control procedures, see HCP quality control documentation (Marcus et al. 2013).

### Voxel-based morphometry pre-processing

Raw T1 data were pre-processed using statistical parametric mapping software (SPM12; Wellcome Department of Cognitive Neurology, London, UK, https://www.fil.ion.ucl.ac.uk/spm/) implemented in MATLAB (Mathworks Inc., Natick, MA). Initially, T1-weighted images were segmented into grey matter, white matter and cerebrospinal fluid using an extension of the standard unified segmentation model in SPM12. The resulting grey matter volumes from the segmentation step were normalised to Montreal Neurological Institute (MNI) standard space generating template images and flow fields. Grey matter volumes were spatially normalised across all participants using the DARTEL algorithm (Ashburner 2007) voxel size: 0.7 × 0.7x 0.7 mm^3^ in MNI space. Finally, the data were smoothed with a 6mm FWHM (full-width half-maximum) Gaussian Filter (Shen and Sterr 2013). Images were then modulated to create Jacobian-scaled grey matter images using deformations estimated in the DARTEL step. Total intracranial volume was calculated by summing the values of grey matter, white matter, and cerebrospinal fluid using the 'tissue volumes' option within SPM12.

### Behavioural data analysis

Win-stay behaviour was categorised as repeating the previous choice following a reward. Lose-shift behaviour was categorised as changing responses from a previous selection following a loss. To assess WSLS behaviour, a paired samples *t-*test comparing behavioural strategies (WSLS behaviour) was conducted on the behavioural task data using SPSS 26 (IBM, SPSS Statistics, Chicago, IL, USA).

### MRI quality check

Quality control procedures were undertaken for the HCP dataset by trained radiologists (see Marcus et al. 2013). We also conducted a thorough quality assessment of the data using tools available in the CAT12 toolbox within the SPM framework (see supplementary document S1 & S2). Preprocessing included automated quality control checks for image homogeneity and noise, as implemented in CAT12. Pre- and post-processing noise values (measured as mean correlation and overall image quality index) were examined to detect images with excessive artefacts or poor signal quality.

The majority of the images (97.3%) fell within the acceptable range of homogeneity, with Z-scores tightly distributed around the median value of approximately 1.3 (considered as excellent image quality by the CAT12 manual). A small number of images appeared as outliers with higher Z-scores (> 2.5), representing less homogeneous data but remaining within an acceptable range for inclusion in the analysis. Overall, the results confirmed excellent to good image quality across the dataset, with the majority of images demonstrating high homogeneity and low noise. A total of 23 outliers with higher Z-scores (between 4 and 5) were visually inspected and retained, as they did not exhibit significant artefacts or distortions that would warrant exclusion.

### VBM analysis

Voxel-based morphometry (VBM) analysis was conducted following established guidelines (Ashburner and Friston 2000; Good et al. 2001). Behavioural measures—the frequency of win-stay and lose-shift behaviours—were entered into a multiple regression model using the “Basic Models” module in SPM12. Covariates included age, sex, and total intracranial volume to account for potential confounding factors. Previous neuroimaging studies have indicated that total GMV decreases linearly with age in normal adult brains, with additional interactions by sex (Good et al. 2001; Ren et al. 2024). Further, we controlled for sex, age, and total GMV, consistent with prior neuroimaging studies (e.g., Ren et al. 2024). To eliminate noisy voxels, we masked the smoothed images with an absolute threshold masking of 0.2 as in previous studies (e.g., Ren et al. 2024). All VBM analyses were performed using SPM12, run under MATLAB software (The Mathworks, Inc., Natick, MA). A cluster size threshold of 5 voxels was selected.

In all regression models, collinearity diagnostics confirmed that multicollinearity was not a concern, with VIF values below 1.1 and Tolerance values exceeding 0.9 for all predictors. These results indicate that the win-stay and lose-shift behavioural variables were sufficiently independent. Consistent with our hypothesis, lose-shift behaviour demonstrated significant negative associations with GMV across the left superior temporal gyrus (LSTG), right middle temporal gyrus (RMTG), and bilateral lateral occipital cortices (Right LOC, Left LOC). In contrast, no significant associations were observed between win-stay behaviour and GMV, suggesting that distinct neuroanatomical mechanisms underpin adaptive decision-making following wins versus losses.

For VBM analysis (Ashburner and Friston 2000; Good et al. 2001), the frequency of ‘stays’ after rewards and frequency of ‘shifts’ after losses were entered into a multiple regression model within SPM12 that controlled for age, sex and total intracranial volume. To control for multiple comparisons, the Family Wise Error (FWE) was set at a threshold of p < 0.05.” We also conducted an exploratory analysis to examine four possible behavioural strategies: 1. Win-stay: Repeating a choice after a reward, 2. Win-shift: Switching a choice after a reward, 3. Lose-stay: Repeating a choice after a loss, and 4. Lose-shift: Switching a choice after a loss. For this analysis, a whole-brain VBM analysis was performed using multiple regression, with all four behavioural strategies entered as predictors. The aim of this follow-up analysis was to determine whether GMV was associated with any additional strategies beyond win-stay and lose-shift behaviour.

## Results

### Random effects testing

Following the approach used by Zhang et al. (2021), we implemented a one-way ANOVA to assess whether participants’ shift and stay behaviours were random (i.e., not influenced by any specific strategy) or systematically affected by the outcome of the previous trial (win or loss). This analysis compared the proportions of four decision strategies: win-stay, win-shift, lose-stay, and lose-shift. The results revealed a significant effect of decision strategy *F*(3,3399) = 63.54, *p* < 0.001, η^2^ = 0.05, indicating that the proportions of these strategies differed significantly. These findings suggest that participants' choices were not random and were systematically influenced by the outcome of the preceding trial, reflecting a structured adaptation rather than random behaviour (Table 1).

**Table 1:** The descriptive statistics for each strategy

Strategy	Mean (%)	SD	SE	Coefficient of variation
Lose-stay	28.08	8.49	0.29	0.30
Lose-shift	22.00	8.41	0.29	0.38
Win-stay	26.81	9.89	0.34	0.37
Win-shift	23.12	9.76	0.34	0.42

### Descriptive statistics

#### Post-hoc pairwise comparisons

We conducted post-hoc Tukey’s HSD tests to examine pairwise differences between the strategies.

**Fig. 2:**
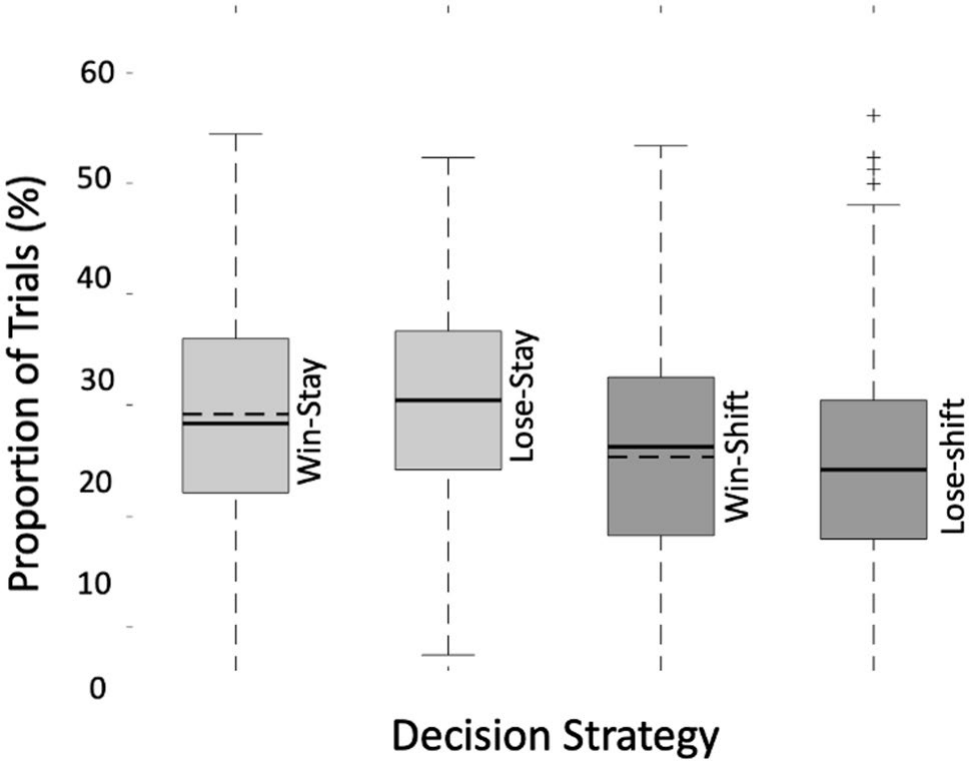
Boxplot depicting the percentage of overall trials within the four decision strategies. The black straight line indicates the median values for each strategy. The black dotted line indicates the mean value for each strategy. For lose-stay and lose-shift behaviour, the median and mean lines overlap. On average, participants employed the Win-Stay strategy in 26.81% of trials, Lose-Stay in 28.06%, Win-Shift in 23.12%, and Lose-Shift in 22.00% of trials

The comparisons in Table 2 confirm that the differences between most strategy pairs are statistically significant, except for Lose-shift vs. Win-Switch, where the adjusted p-values are marginally above the threshold for significance (Fig. 2).

**Table 2:** Post-hoc Tukey’s HSD test to examine pairwise differences between each of the four strategies

Comparison	Mean difference	SE	t	p (Tukey)
Lose-stay vs. lose-shift	6.08	0.44	13.68	< .001
Lose-stay vs. win-stay	1.27	0.44	2.86	0.022
Lose-stay vs. win-shift	4.97	0.44	11.17	< .001
Lose-shift vs. win-stay	−4.81	0.44	−10.82	< .001
Lose-shift vs. win-shift	−1.12	0.44	−2.51	0.058
Win-stay vs. win-shift	3.69	0.44	8.31	< .001

### Behavioural analysis

In our study, we initially focused on two main behavioural conditions: 1. The frequency of win-stay behaviour (repeating a choice following a reward), and 2. The frequency of lose-shift behaviour (switching a choice following a loss). To further examine the relative dominance of different decision strategies and explore participants’ choice behaviour to prior outcomes, we conducted a series of pairwise comparisons using paired-sample t-tests. Participants tended to use the win-stay strategy (M = 54.53% SD = 4.89) more than the lose-shift strategy (M = 45.47% SD = 4.11; *t(*850) = 10.88, *p* < 0.001). Participants also tended to use the lose-stay strategy (M = 55.57%, SD = 15.54), more that the win-shift strategy (M = 44.43%, SD = 15.54; *t(*850) = 10.44, *p* < 0.001). When comparing strategies following win outcomes, participants tended to use the win-stay strategy (M = 53.65%, SD = 19.56) more than a win-shift strategy (M = 46.35%, SD = 19.56, *t(*850) = 4.44, *p* < 0.001). Following loss outcomes, participants tended to use the lose-stay strategy (M = 56.03%, SD = 16.72) more than the lose-shift strategy (M = 43.97%, SD = 16.73, *t(*850) = 10.47, *p* < 0.001).

### GMV VBM analysis

**Fig. 3:**
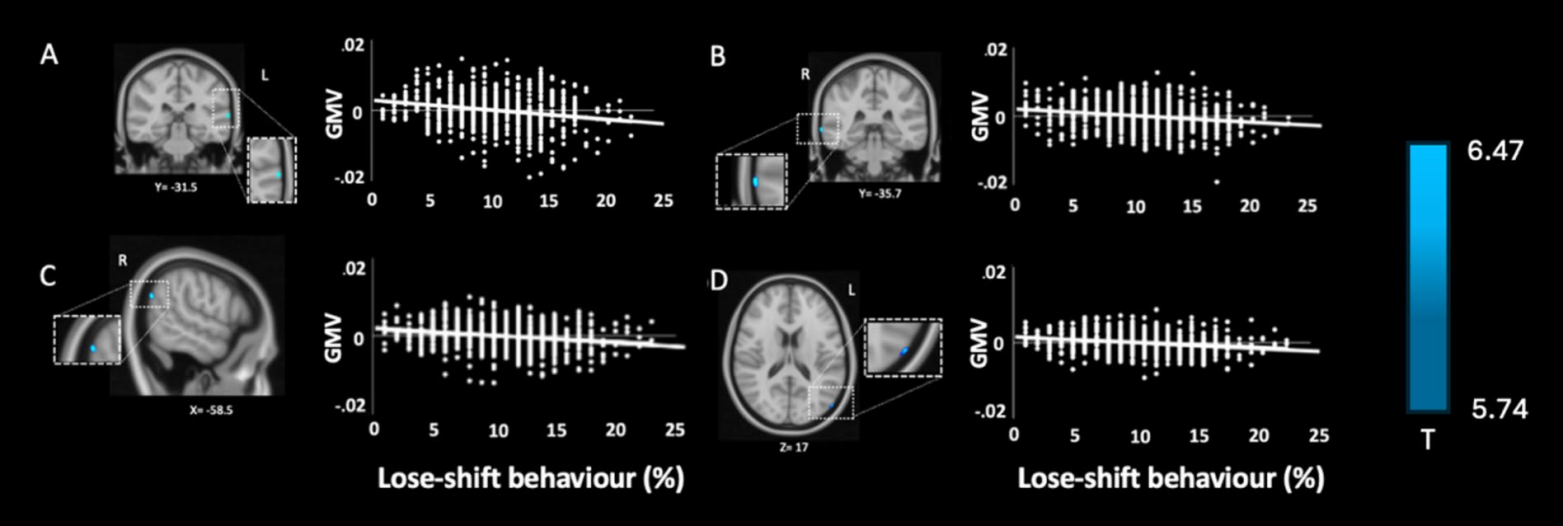
Whole brain statistical maps in MNI space showing negative correlations between GMV and lose-shift behaviour. Slices were chosen to best display the area of interest alongside plots comparing GMV against lose-shift behaviour. FWE = .05. **A**: Coronal view of left STG (r = −0.04, p < .001). **B:** Coronal view of right MTG (r = −0.03, p < .001). **C:** Sagittal view of right SLOC (r = −0.04, p < .001). **D:** Axial view of left SLOC (r = −0.03, p < .001). Slices were chosen to clearly identify brain regions associated with lose-shift behaviour. T maps are shown in radiological inversion

Whole brain corrected VBM analysis (FWE = 0.05) using multiple regression was conducted to assess whether GMV was associated with each strategy (win-stay, win-shift, lose-stay, lose-shift). As previous studies have indicated that total GMV decreases linearly with age in normal adult brains, and interacts with sex (Good et al. 2001; Ren et al. 2024), we controlled for sex, age, and total GMV. No clusters were associated with lose-stay, win-stay or win-shift behaviour. However, lose-shift behaviour was negatively correlated with GMV in the left superior temporal gyrus (STG), right middle temporal gyrus (MTG) and bilateral occipital cortices (see Fig. 3). Correlation analyses revealed significant but small negative associations between GMV and lose-shift behaviour across key regions (r = correlation coefficient,). The left superior temporal gyrus (STG; r = − 0.04, *p* < 0.001) and right superior lateral occipital cortex (SLOC; r = − 0.04, *p* < 0.001) explained 0.16% of the variance in lose-shift behaviour. Similarly, the right middle temporal gyrus (MTG; r = − 0.03, *p* < 0.001) and left SLOC (r = − 0.03, *p* < 0.001) explained 0.09% of the variance. Collinearity analyses were conducted to ensure the independence of the predictors in the regression models. Variance inflation factor (VIF) values for all predictors—age, total intracranial volume (TIV), win-stay, and lose-shift behaviours—remained below the threshold of 5 (range: 1.014–1.090), indicating no substantial collinearity. Tolerance values similarly supported the independence of the regressors (range: 0.918–0.986) (Table 3).

**Table 3:** Results from the whole-brain VBM multiple regression showing a negative correlation with lose-shift behaviour (FWE = .05)

Region	Cluster size (mm^3^)	Side	Peak T value	X	Y	Z	p
Superior temporal gyrus	79.96	Left	6.47	−66.1	−31.5	7.1	< .001
Middle temporal gyrus	104.96	Right	6.00	71.8	−35.7	−1.5	< .001
Superior lateral occipital cortex	68.94	Right	5.95	58.5	−64.4	27	< .001
Superior lateral occipital cortex	14.06	Left	5.74	−52.8	−78.4	17	< .001

### WMV VBM analysis

**Fig. 4:**
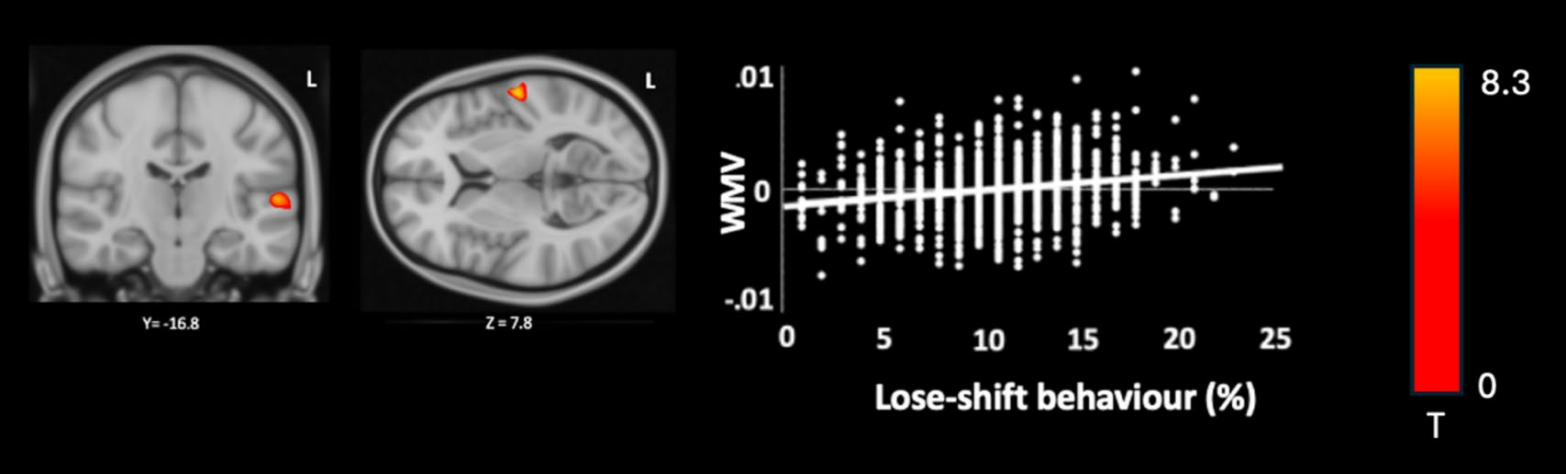
Figure in MNI space illustrating increases in WMV within the left superior temporal gyrus associated with lose-shift behaviour. T-map is shown in radiological inversion

Whole-brain white matter VBM multiple regression (FWE = 0.05) showing a positive correlation (Z = 5.83, cluster size = 1137, MNI coordinates: x = −58.4, y = −16.8, T = 8.3, p < 0.001) between WMV in the STG and lose-shift behaviour. No clusters were associated with win-stay, win-shift, or lose-stay behaviour (Fig. 4).

## Discussion

In the current study, we explored the relationship between brain morphometry and switching behaviour, with a focus on the use of the Win-stay, Lose-shift (WSLS) strategy. In our first hypothesis, we predicted that there would be a significant positive correlation between GMV in the frontal pole, posterior cingulate and left insula and the overall tendency to switch response options irrespective of the outcome of the previous trial. In our second hypothesis, it was predicted that there would be a significant negative correlation between GMV of the MTL and right insula and the overall tendency to switch response options irrespective of the outcome of the previous trial. Contrary to these hypotheses, we found no significant associations between GMV or WMV and the overall tendency to switch response options, irrespective of the outcome of the previous trial. Finally, it was hypothesised that lose-shift behaviour would be associated with GMV of the right insula and right MTL. Contrary to our hypothesis, we found that lose-shift behaviour was associated with reduced GMV within the right STG, left MTG and bilateral SLOC. In addition, lose-shift behaviour was positively correlated with WMV within the left STG. No correlations between win-stay and brain morphometry survived correction. This is, to the authors’ knowledge, the first time that lose-shift behaviour has been linked with reduced GMV and increased WMV.

Our findings broaden the understanding of approach-avoidance systems, including WSLS behaviour, by emphasising the involvement of brain regions that are not traditionally associated with this framework, such as the STG, MTG, and SLOC. While core regions related to approach-avoidance, such as the striatum, insula, anterior cingulate cortex and prefrontal cortices, drive immediate reward-seeking or loss-avoidance behaviours (Livermore et al. 2021; Zorowitz et al. 2019), the STG appears to play a key role in contextual outcome processing and integration of negative feedback to guide subsequent decision-making (Paulus et al. 2005). Similarly, reduced GMV in the MTG may impair working memory-dependent mechanisms, such as retaining and updating prior outcome information, which are essential for flexible behavioural adjustments (Foerde et al. 2013). Indeed, several studies have implicated neighbouring regions of the STG, such as the supramarginal gyrus (Canessa et al. 2017), posterior insula (Canessa et al. 2013; Markett et al. 2016), and opercular cortex (Canessa et al. 2022), in processes related to loss aversion. While it is possible that shared functions related to loss aversion may influence the findings of this study, it is crucial to exercise caution when interpreting the involvement of neighbouring brain regions. Spatial proximity does not necessarily imply a shared function (Alexander-Bloch et al. 2013; Mišić et al. 2014) and further research is needed to delineate the specific contributions of these regions to loss aversion and related cognitive processes. Future work could benefit from higher-resolution imaging to disentangle these closely situated regions and further clarify their specific roles in the context of loss aversion. Finally, reductions in GMV in the occipital cortex align with recent evidence suggesting its role in processing visual uncertainty cues and outcome salience (Zhang et al. 2024). These findings indicate that loss-shift behaviour, rather than solely resulting from reward-punishment systems, may involve a more complex interaction of contextual evaluation, memory integration, and uncertainty monitoring. By identifying structural correlates in these regions, our results expand current models of adaptive learning and loss-related decision-making.

The STG and MTG findings align with established frameworks of feedback-based learning and contextual processing. The STG has been implicated in integrating negative feedback and contextual cues to guide adaptive decision-making, suggesting that reduced GMV may reflect diminished neural resources for responding to losses (Paulus et al. 2005). Similarly, the MTG plays a critical role in working memory modulation and retaining outcome history, processes necessary for flexible behavioural adjustment following negative outcomes (Foerde et al. 2013). Specifically, lose-shift behaviour may engage processes of aversive response modulation and goal-directed decision-making, which are potentially linked to regions such as the STG and MTG. These regions have been implicated in processing salience and integrating multimodal information, supporting their role in associating the context of a negative outcome with adaptive shifts in subsequent behaviour (Menon and Uddin 2010; Visser et al. 2012; Humphreys et al. 2015; Zou et al. 2024). In contrast, the lack of significant findings for win-stay behaviour underscores the possibility of distinct neural mechanisms driving responses to positive feedback compared to negative feedback, suggesting that the two types of strategy may rely on fundamentally different cognitive and neural pathways (Kahneman 2011). Furthermore, the association between reduced GMV in the occipital cortex and lose-shift behaviour aligns with its role in processing visual salience and uncertainty cues, which are essential for recognising and adapting to unfavourable outcomes (Zhang et al. 2024). These findings expand traditional approach-avoidance models, which focus predominantly on regions such as the striatum, insula, anterior cingulate cortex and prefrontal cortices (Livermore et al. 2021; Zorowitz et al. 2019). We suggest that lose-shift behaviour reflects an interaction of contextual evaluation (STG), memory-based updating (MTG), and uncertainty monitoring (occipital cortex), highlighting a broader neural basis for adaptive feedback-based strategies.

The role of adaptive behavioural learning in this task warrants careful consideration. Adaptive behavioural learning is a core mechanism underpinning behavioural flexibility and the neural processes that support decision-making in dynamic environments (O’Reilly 2013; Schulz et al. 2019). Notably, paradigms investigating adaptive behavioural learning often focus on the development of strategies aimed at achieving optimal decision-making outcomes (e.g., Trimmer et al. 2015; Schiffer et al. 2017; Sharif et al. 2024). However, the task employed in this study lacks a clearly defined optimal decision-making strategy, which is needed to support adaptive behavioural learning. As such, while it is plausible that the task engages some neural mechanisms similar to those involved in adaptive behavioural learning, it should also be interpreted in the context of its design, which does not necessitate learning of optimal outcomes.

While lose-shift behaviour was associated with both GMV and WMV, the current study found no such association with win-stay behaviour, suggesting that its neural underpinnings may not be directly related to brain morphology. This raises the possibility that win-stay strategies could involve more complex neural interactions or functional activations not captured by structural volumetric measures. Supporting this idea, Van de Steen et al. (2020) found that during the same task, increased activation in the occipital cortex was linked to win and loss outcomes but not to neutral trials, implying that specific neural activations are more responsive to outcome valence. This finding suggests that win-stay strategies likely depend on dynamic brain responses to trial-by-trial win outcomes, whereas there is evidence that responses to losses can remain more stable across tasks (see Spektor et al. 2024). Further, activation in the occipital cortex has been associated with processing visual cues related to uncertainty and free energy (Zhang et al. 2024). In the “Free energy” principle proposed by Friston (2009), the brain is thought to attempt to minimise “free energy,” which is a measure of the difference between top-down predictions and actual sensory inputs, to enable the agent to adapt to their environment (Friston 2009). This explanation may account for the association between reduced GMV in the occipital cortices and the increased frequency of 'lose-shift' behaviour. Specifically, individuals with lower GMV in these areas may struggle more with adapting to loss outcomes, making them more likely to switch strategies after a negative outcome. Additionally, individual differences in sensitivity to losses (Adrián-Ventura et al. 2019) or uncertainty (Zhang et al. 2024) may influence decision-making in the task used in the current study, as it may increase the drive to minimise prediction errors, resulting in more frequent lose-shift behaviour. Given the stability of individual differences in responses to losses across different task types (Spektor et al. 2024), future longitudinal research should examine how lose-shift behaviour in this task relates to broader measures of loss aversion.

Contrary to our hypotheses, and the findings by Sun et al. (2018), we did not find an association between overall response-switching and GMV regardless of the outcome of the previous trial. It is unclear what might have caused this discrepancy as although the tasks were different, they elicited similar percentages of switch responses (current study: 40%, Sun et al. 2018: 43%). One intriguing possibility might be that the findings reflect cultural differences between US and Asian populations in how rewards and losses are processed (e.g., Chen et al. 2020). However, further research would be required to substantiate this. Alternatively, these different findings might be due to the disparity in ages between the two studies (average age: 28.7 years, Sun et al.’s (2018) average age was 19.9) as prior evidence suggests that grey matter volume changes with age (Bourisly et al. 2015; Hafkemeijer et al. 2014; Fjell and Walhovd 2010; Resnick et al. 2003).

Several limitations of this study should be acknowledged. First, the task was presented in blocks of eight trials that were either mostly reward or mostly loss, but still contained a mix of trial types. The organisation of these blocks may have influenced participants by making rewards and losses less or more impactful, thus influencing choices throughout the experiment. In blocks where losses predominated, participants may have experienced a heightened sensitivity to losses, potentially increasing the likelihood of lose-shift behaviour as they adjusted their choices in response to the negative outcomes. Conversely, in blocks where rewards were more frequent, the impact of occasional losses might have been diminished as a win is two times the value of a loss, possibly leading to a reduced tendency to shift behaviour after a loss.

Second, reduced GMV in the temporal and occipital cortices associated with lose-shift behaviour may not directly reflect the neuroanatomical basis for loss aversion, or impairments in attention or memory processing, particularly given the absence of an optimal decision-making strategy in the task. While these effects are statistically significant due to the large sample size, the small proportion of explained variance suggests subtle structural contributions to lose-shift behaviour. To better understand this relationship, future studies should investigate how brain morphometry is associated with WSLS behaviour in tasks that include clear optimal decision strategies. This could help determine whether variations in WSLS behaviour contribute to maladaptive decision-making. While factors such as outcome history and risk preferences are known to influence decision-making (Sitkin and Weingart 1995), these parameters were not included in the current analysis. The forced-choice card-guessing task used in this study lacked a clearly defined or optimal decision strategy, making it less suited to capturing risk propensity, adaptive decision making or learning. Instead, the task encouraged heuristic responses, such as WSLS behaviours. Moreover, including additional parameters could have introduced unnecessary complexity and confounded the interpretation of our results, particularly as these behaviours are not inherently tied to explicit risk-reward structures. Thirdly, it should be noted that the associations we found were correlational, so it remains unclear whether behaviour was the consequence or the cause of the variation in GMV. Finally, while the use of VBM to analyse WMV has faced scrutiny due to concerns regarding segmentation accuracy and partial volume effects (Ashburner 2007), these limitations have been addressed through advancements in preprocessing pipelines. In particular, the DARTEL algorithm used in our study improves normalisation accuracy, enhancing sensitivity to subtle volumetric differences (Ashburner 2007). Additionally, by controlling for TIV we minimised confounds associated with individual differences in brain size, further strengthening the validity of our results (Whitwell 2009). Our inclusion of WMV aligns with literature emphasising its role in structural connectivity and its contributions to cognitive and behavioural processes. For instance, WMV reductions have been linked to age-related visual changes in eye disease (Hernowo et al. 2014), theory of mind abilities (Soylu et al. 2023), and cognitive impairments in conditions such as early-onset psychosis (Si et al. 2024) and Alzheimer’s disease (Li et al. 2012). These studies derived WMV through VBM demonstrating that WMV variations can serve as meaningful markers of brain-behaviour relationships across domains. Future research should replicate these results with the same sample using combined metrics of WMV and diffusion tensor imaging, as in Michel et al. (2024) to further understand the contributions of white matter in behavioural choice adaptations. Furthermore, future studies could explore how structural findings interact with functional activations during feedback-based tasks, providing a more comprehensive understanding of the neural basis of win-stay and lose-shift behaviour.

In summary, this study provides evidence that lose-shift behaviour is associated with reductions in GMV within the STG, MTG, and bilateral occipital cortex and a reduction in WMV in the STG. These results may be explained through the roles of specific brain regions, such as the occipital cortices, in directing attention to task-relevant stimuli, which, in turn, impact lose-shift behaviour. Further research is required to more systematically examine the precise role each brain region plays in decision-making processes.

## Supplementary Information

Below is the link to the electronic supplementary material.

Supplementary file1 (DOCX 399 KB)

## Data Availability

No datasets were generated or analysed during the current study.
