# Supplementary material for: Examining neuroanatomical correlates of win-stay, lose-shift behaviour
Source: Brain Struct Funct. 2025 Feb 27;230(2):40. doi: 10.1007/s00429-025-02901-z (PMC11868257; doi:10.1007/s00429-025-02901-z)

Supplementary Information

S1: Post-processing noise values. This image illustrates the noise values observed following the application of CAT12 data quality check. The scatter plot revealed that most scans had Z-scores between 1 and 2, indicating high homogeneity. A small number of scans exhibited Z-scores > 2.5 (up to ~4.5), flagged in the yellow-to-red range. We have visually inspected the flagged outliers using the "Check Worst" option and these scans do not exhibit artefacts or abnormalities that warrant exclusion. This confirms the suitability of all included processed T1-weighted MRI scans for subsequent analyses.


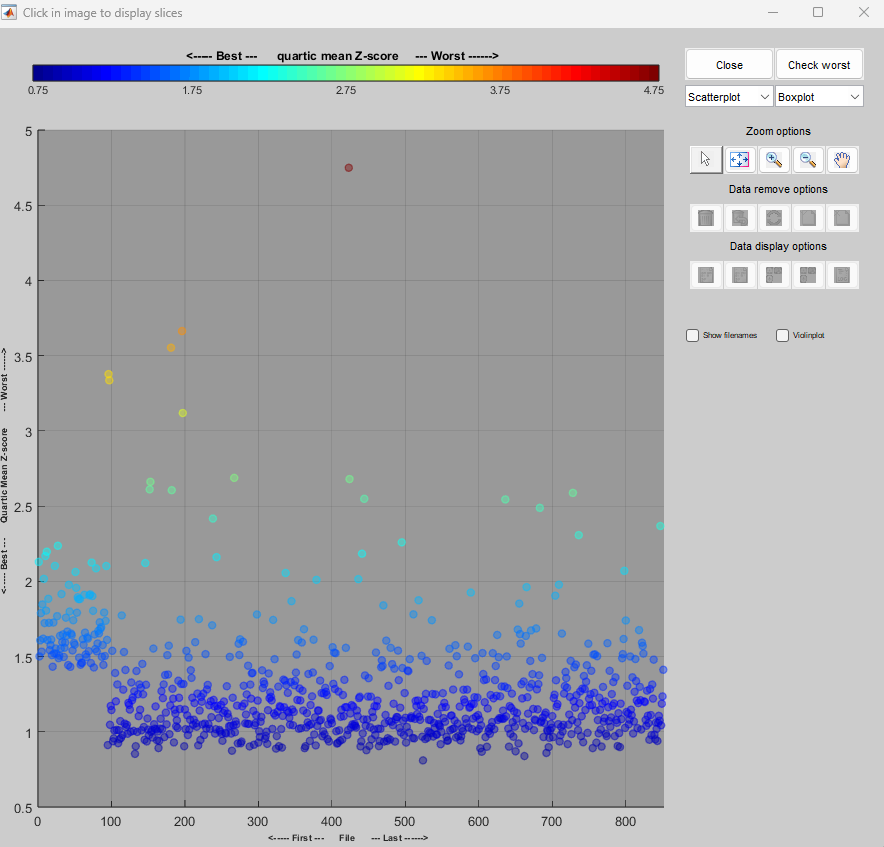


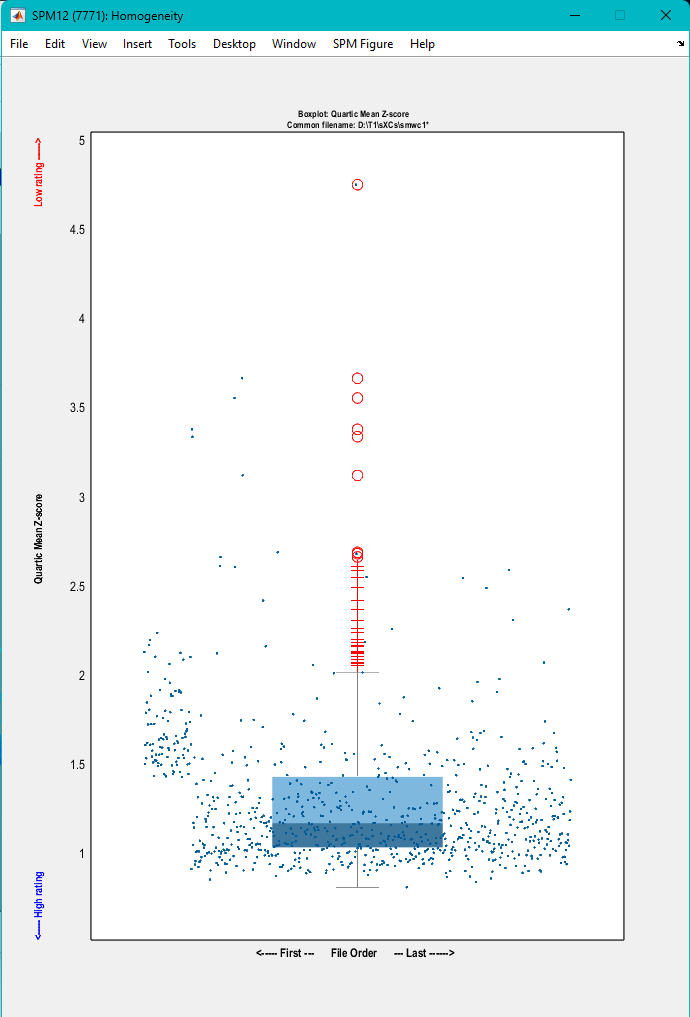


S2: Pre-processing noise values. The majority of scans are concentrated in the blue-to-green range, indicating good quality and minimal deviations, while a few points in the yellow-to-red range suggest higher Z-scores, potentially indicating artefacts or anomalies. To ensure the integrity of the dataset, all scans flagged by this QC analysis were visually inspected for artefacts using the “Check Worst” function. No significant issues were identified, and no scans were removed from the dataset. This confirms the suitability of all included raw T1-weighted MRI scans for further processing.


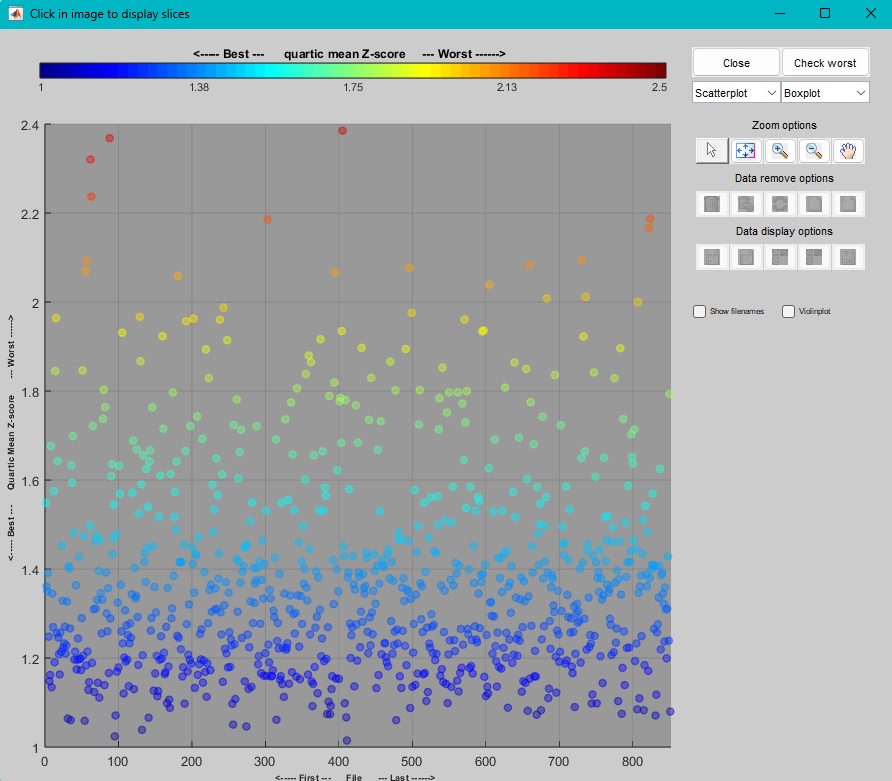


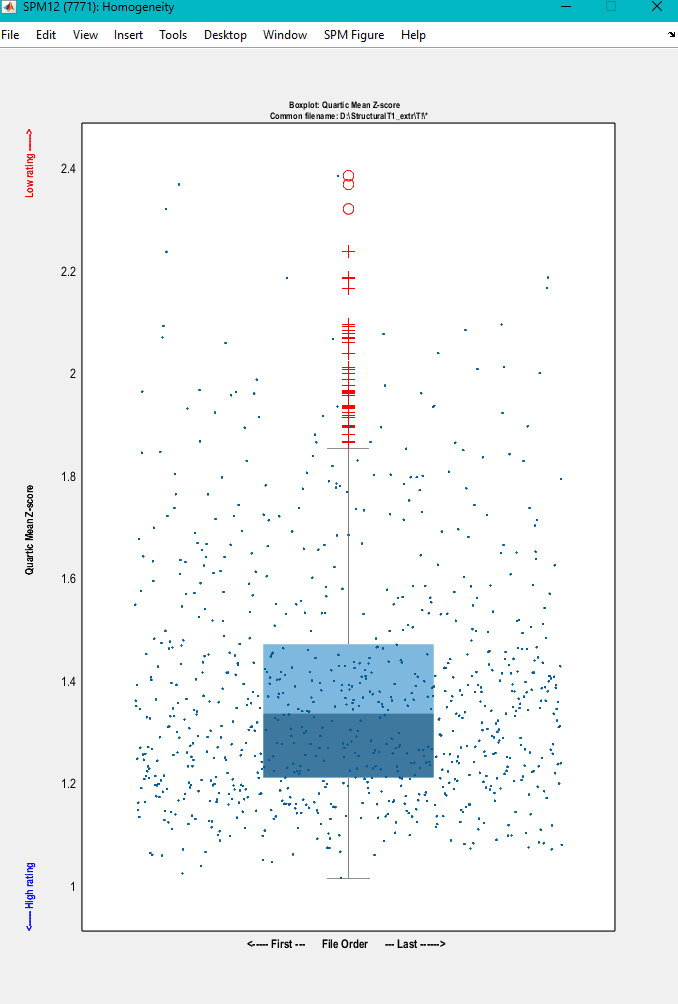

Supplement: Supplementary file 1 — Supplementary file1 (DOCX 399 KB) [file 429_2025_2901_MOESM1_ESM.docx]
